# *Aspergillus fumigatus* Fumagillin Contributes to Host Cell Damage

**DOI:** 10.3390/jof7110936

**Published:** 2021-11-03

**Authors:** Xabier Guruceaga, Uxue Perez-Cuesta, Aize Pellon, Saioa Cendon-Sanchez, Eduardo Pelegri-Martinez, Oskar Gonzalez, Fernando Luis Hernando, Emilio Mayayo, Juan Anguita, Rosa M. Alonso, Nancy P. Keller, Andoni Ramirez-Garcia, Aitor Rementeria

**Affiliations:** 1 Fungal and Bacterial Biomics Research Group, Department of Immunology, Microbiology and Parasitology, Faculty of Science and Technology, University of the Basque Country (UPV/EHU), 48940 Leioa, Spain; xabier.guruceaga@ehu.eus (X.G.); uxue.perezc@ehu.eus (U.P.-C.); saioa.cendon@ehu.eus (S.C.-S.); eduardo.pelegri@ehu.eus (E.P.-M.); fl.hernando@ehu.eus (F.L.H.); 2 Inflammation and Macrophage Plasticity Laboratory, CIC bioGUNE-BRTA (Basque Research and Technology Alliance), 48160 Derio, Spain; aize.pellon@kcl.ac.uk (A.P.); janguita@cicbiogune.es (J.A.); 3 FARMARTEM Group, Department of Analytical Chemistry, Faculty of Science and Technology, University of the Basque Country (UPV/EHU), 48940 Leioa, Spain; oskar.gonzalezm@ehu.eus (O.G.); rosamaria.alonso@ehu.eus (R.M.A.); 4 Pathology Unit, Medicine and Health Science Faculty, University of Rovira i Virgili, 43201 Reus, Spain; emilio.mayayo@urv.cat; 5 Ikerbasque, Basque Foundation for Science, 48011 Bilbao, Spain; 6 Department of Medical Microbiology and Immunology, University of Wisconsin, Madison, WI 53706, USA; npkeller@wisc.edu; 7 Department of Bacteriology, University of Wisconsin, Madison, WI 53706, USA

**Keywords:** fumagillin, *Aspergillus fumigatus*, virulence factor, pathogenesis, RAW 264.7, A549, mice infection, UHPLC

## Abstract

The activity of fumagillin, a mycotoxin produced by *Aspergillus fumigatus*, has not been studied in depth. In this study, we used a commercial fumagillin on cultures of two cell types (A549 pneumocytes and RAW 264.7 macrophages). This toxin joins its target, MetAP2 protein, inside cells and, as a result, significantly reduces the electron chain activity, the migration, and the proliferation ability on the A549 cells, or affects the viability and proliferation ability of the RAW 264.7 macrophages. However, the toxin stimulates the germination and double branch hypha production of fungal cultures, pointing out an intrinsic resistant mechanism to fumagillin of fungal strains. In this study, we also used a fumagillin non-producer *A. fumigatus* strain (∆*fmaA*) as well as its complemented strain (∆*fmaA::fmaA)* and we tested the fumagillin secretion of the fungal strains using an Ultra High-Performance Liquid Chromatography (UHPLC) method. Furthermore, fumagillin seems to protect the fungus against phagocytosis in vitro, and during in vivo studies using infection of immunosuppressed mice, a lower fungal burden in the lungs of mice infected with the ∆*fmaA* mutant was demonstrated.

## 1. Introduction

*Aspergillus fumigatus* is a saprophytic fungus that can colonize diverse ecological niches facilitated by its great plasticity to adapt to different environments and the wide dispersal of its small, airborne spores called conidia [1,2]. The conidia allow *A. fumigatus* to reach all chambers of the human respiratory tract, where the fungus is able to cause a wide range of infections depending on the immune status of the patient [3,4,5,6].

The biological characteristics that allow *A. fumigatus* to colonize and/or infect the respiratory tract are known as virulence factors. These include the fungal cell wall and the proteins involved in its formation, the resistance of the conidia to ultraviolet radiation and dryness, the mechanisms to evade the immune response, and the production of proteases and toxins [6]. In fact, the *A. fumigatus* genome contains a great variety of biosynthetic clusters [7,8] that provide the fungus with complex and bioactive secondary metabolites, including the production of toxins [9,10,11]. Among the variety of toxins produced by *A. fumigatus*, several have been linked with enhanced virulence and include gliotoxin [12,13,14,15], fumitremorgin A and B [16,17], hexadehydroastechrome [18], hemolysin, and mitogillin [19].

The role of fumagillin in fungal virulence has not been completely elucidated yet. This toxin has been widely studied for other reasons, such as tumor control due to the inhibition of endothelial cell proliferation and its effect as an anti-tumor molecule [20]. Furthermore, it has been used as an antibiotic agent in the treatment against different pathogens, such as microsporidia, parasites, and as a treatment of diseases such as AIDS and obesity [21]. The mycotoxin is produced by a series of enzymes encoded in a biosynthetic cluster located on chromosome 8 of *A. fumigatus* [22]. The members of the biosynthetic cluster are overexpressed in the lungs of intranasally infected mice [23] and include the fumagillin pathway-specific transcription factor [24]. In fact, a non-fumagillin producer mutant strain caused significantly less cellular damage than the wild type in vitro, demonstrating a potential relevance of this toxin in virulence [23].

Given these previous data and the need to deeply analyze the involvement of secondary metabolites in virulence [25], the aim of the present study was to assess the role of fumagillin in the pathogenesis of *A. fumigatus*. For this purpose, we studied the effect of commercial fumagillin on *A. fumigatus* itself, macrophages, and epithelial cells. Additionally, a non-fumagillin producer mutant strain was used in co-incubation with cell cultures and during murine infection to determine the potential impact of the toxin on fungal virulence.

## 2. Materials and Methods

### 2.1. Aspergillus fumigatus Strains, Media, and Growth Conditions

The Δ*akuB*^*ku80*^ strain of *A. fumigatus* was used as a wild-type (Wt) strain during this study. Furthermore, we used the previously published [22] deletion mutant strain ∆*fmaA* (a non-fumagillin producer strain) and its complemented strain ∆*fmaA*::*fmaA* [23]. All the strains were grown on glucose minimal medium agar (GMM) for seven days at 37 °C, as previously described [26]. Conidia were harvested and cleaned twice with saline-Tween solution (SS-T; 0.9% NaCl and 0.02% Tween 20). The number of conidia for each experiment was adjusted using a Bürker counting chamber.

### 2.2. Aspergillus fumigatus Phenotypic Characterization

The phenotypes of the mutant *A. fumigatus* strains used in this work were evaluated using the spot dilution assay protocol, as previously described [27]. Briefly, we supplemented GMM medium with 80 µg/mL congo red (CR), 40 µg/mL calcofluor white (CW) or 0.0125% sodium dodecyl sulfate (SDS) as cell wall/membrane stress agents; 1 M NaCl, 1 M KCl, or 1.2 M sorbitol as osmotic stress agents; or hydrogen peroxide (30%, 15%, 7.5%, and 3.75% *v*/*v*) as an oxidative stress agent.

### 2.3. Cell Lines

Two different cell lines obtained from the American Type Culture Collection (ATCC, Manassas, VA, USA) were used in this study: the murine macrophage cell line RAW 264.7 and the human alveolar epithelial cell line A549. Cell cultures were maintained in RPMI 1640 medium supplemented with 10% heat-inactivated fetal bovine serum (FBS), 200 mM l-glutamine, 100 U/mL penicillin, and 0.1 mg/mL streptomycin (complete RPMI), and incubated at 37 °C, 5% CO_2_, and 95% of humidity (cell culture atmosphere). Cell line passages were used only when the viability of the cells was higher than 90%. All culture media components were from Sigma–Aldrich (St. Louis, MO, USA).

### 2.4. Isolation and Culture of Mouse Primary Macrophages

Mouse bone marrow-derived macrophages (BMMs) were generated following the method previously published using 8–12 week-old C57Bl/6 mice [28]. Briefly, bone marrow material was obtained by flushing mouse femurs and tibias in complete RPMI. The cell suspensions were filtered through a 70 µm-nylon mesh (ThermoFisher, Waltham, MA, USA) and then centrifuged at 400 rpm for 5 min. Then, ammonium–chloride–potassium (ACK) lysis buffer was used to remove red blood cells. The remaining cells were incubated in untreated 100 mm × 15 mm Petri dishes for 7 days in the presence of complete RPMI supplemented with 30 ng/mL of M-CSF (Miltenyi Biotec, Bergisch Gladbach, Germany). Fresh medium was added after 3 days of culture.

### 2.5. Quantification of Fumagillin Secretion by Aspergillus fumigatus

To study the ability of each fungal strain to produce fumagillin, we seeded 5 × 10^6^ conidia/mL of each strain in 2 mL of complete RPMI using 6-well plates (ThermoFisher). The plates were incubated at 37 °C, 5% CO_2_, and 95% humidity. After 48 h of incubation, we centrifuged an aliquot of 1 mL at 14,000 rpm for 5 min, and the supernatant was transferred to a light-safe microtube and kept on ice until its measurement by UHPLC. Each assay was done using four independent conidia batches of each strain harvested just before the assay start point.

### 2.6. Fungal Response to Increasing Concentrations of Fumagillin

To study if fumagillin affects fungal strains, we performed an 8 h germination study in the presence of 0.5, 1, and 2 µg/mL of fumagillin. We seeded 2 × 10^6^ conidia/well using 24 cell culture well plates. In each case, we calculated fungal germination and hyphal branching using a Nikon Eclipse TE2000-U inverted microscope.

Growth curves of the fungal strains were assessed for 70 h in 96-well plates (ThermoFisher, Waltham, MA, USA). We seeded 5 × 10^5^ fresh conidia per well in 150 µL of MM supplemented with 0.5, 1, and 2 µg/mL of fumagillin. The plate was incubated in the microplate reader Synergy^TM^ HT (BioTek, Winooski, VT, USA) at 37 °C, and automatic absorbance measures at 600 nm were done each 30 min for 70 h.

### 2.7. Fumagillin Absorption Ability by Cell Lines

To study the absorption of fumagillin by mammalian cells, we seeded 1 × 10^6^ cells/mL of the RAW 264.7 and A549 cell lines in a final volume of 2 mL of complete RPMI using 6-well plates. After 1 h of incubation to allow cell attachment, we replaced the medium with 2 mL of complete RPMI supplemented with 1 µg/mL of fumagillin (Sigma–Aldrich). Aliquots of 500 µL were collected after 8, 20, and 24 h of cell exposition in light-safe microtubes and processed as previously indicated until its measurement by UHPLC (Waters, Milford, CT, USA).

In order to study whether fumagillin binds to the cell line instead of being degraded, we grew RAW 264.7 and A549 cells in the presence of 2 µg/mL of toxin. After 24 h of exposition, we discarded the supernatants and, after a saline solution wash, we lysed the cells using 1 mL of RIPA buffer. Finally, we centrifuged the samples at 14,000 rpm for 5 min, and the supernatants were measured using the UHPLC method. A toxin degradation study was done by exposition of a 1 µg/mL fumagillin solution to different environmental conditions as 80 °C or exposition to light for 96 h and exposition to pH 1.

### 2.8. Quantification of Fumagillin by UHPLC (Ultra High-Performance Liquid Chromatography) in RPMI Samples

Quantitative analysis of fumagillin was carried out using an Acquity Ultra-High-Performance Liquid Chromatography (UHPLC) system (Waters, Milford, CT, USA) coupled to a photodiode array (PDA) detector, following a methodology previously published [29]. The chromatographic separation was performed on an Acquity BEH C18 column (2.1 mm × 50 mm, 1.7 μm) from Waters. The mobile phases used were a 10 mM ammonia/ammonium buffer (pH 10) as the aqueous mobile phase (A) and acetonitrile as the organic modifier (B). A flow rate of 0.40 mL/min was used with an elution gradient as follows: 0–0.5 min, 20% B; 0.5–5.5 min, linear change from 20% to 95% B; 5.5–6.5 min, 95% B; 6.5–7.0 min, from 95% to 20%. During the chromatographic analysis, the column was maintained at 35 °C with a thermostat, and the samples were kept at 4 °C in the autosampler. Wavelengths of 336 and 280 nm were employed for fumagillin and diclofenac (internal standard, IS), respectively. System control, data collection, and data processing were accomplished using Empower 2 software.

Prior to the chromatographic analysis, a solid-phase extraction (SPE) procedure was applied to the RPMI samples. Each 500 μL RPMI sample was spiked with 25 μL of 20 mg/mL IS solution in methanol and 475 μL of phosphate buffer (100 mM, pH 12). After vortex mixing, the solution was transferred to Oasis MAX cartridges (30 mg, 1 cm^3^) from Waters. The cartridges had been previously activated with 1 mL methanol and conditioned with 1 mL phosphate buffer (100 mM, pH 12). After sample loading, the cartridges were washed with 1 mL phosphate buffer (100 mM, pH 12):methanol (55:45) followed by 5 min drying at high vacuum, and then 1 mL of 3.5% formic acid solution in methanol was used for eluting the analyte. Subsequently, 500 μL of aqueous mobile phase was added to 500 μL of the eluate, and after centrifugation, the solution was transferred to autosampler vials and injected into the UHPLC system for analysis. For the quantification of fumagillin, a calibration curve in RPMI was built with a linear range between 25 and 1500 μg/L.

### 2.9. Cellular Electron Transport Chain Activity

The electron transport chain (ETC) activity of cells in contact with different concentrations of fumagillin was measured using an MTT assay. Briefly, we seeded 8 × 10^4^ cells per well (RAW 264.7 or A549) in 96-well plates (ThermoFisher) with 150 µL of complete RPMI. After 2 h of incubation to allow cell attachment, we replaced the complete RPMI with 150 µL of RPMI supplemented with different concentrations of fumagillin (0, 0.5, 1, and 2 µg/mL), and the cells were incubated using the abovementioned conditions of cell culture atmosphere. After 24 h of incubation, we eliminated the medium, and we added 150 µL/well of complete RPMI supplemented with 0.5 mg/mL of MTT (Sigma–Aldrich) and the plate was incubated for 4 h to allow the reduction of the tetrazolium dye MTT to formazan crystals. Finally, we replaced MTT solution with 150 µL of DMSO in order to dissolve formazan crystals, and after 15 min, the absorbance was measured at 560 nm using a microplate reader Synergy^TM^ HT (BioTek, Winooski, VT, USA). Results were expressed as a percentage of metabolic activity.

### 2.10. Wound Healing Assay

In order to evaluate the growth inhibitory capacity of fumagillin over A549 epithelial cells, we performed wound healing assays. Briefly, 1.5 × 10^5^ cells per well were seeded in 24-well plates and, after an incubation of 24 h, confluent monolayers were obtained. Using a 1000 µL pipette tip, a vertical and a horizontal scratch was made and then washed twice with PBS to remove detached cells. Then, fresh medium alone or containing 0.5, 1, or 2 µg/mL of fumagillin was added to the cultures. Images were taken from one field of view down between the intersection of the two scratches at 24 and 48 h after fumagillin addition, using a Nikon Eclipse TE2000-U (Nikon, Minato, Tokyo, Japan) inverted microscope. Finally, the percentage of scratch reduction was calculated and normalized to the untreated control.

### 2.11. Analysis of Cell Proliferation and Viability by Flow Cytometry

Flow cytometry-based assays were carried out in the general services from the University of the Basque Country (SGIker, UPV/EHU) using a Beckman Coulter Gallios cytometer (Beckman Coulter, Brea, CA, USA).

Changes in cell proliferation of A549 and RAW 264.7 cells in the presence of fumagillin at different concentrations were determined using the fluorescent dye CFSE (Invitrogen, Carlsbad, CA, USA). Cells were stained using 2 µM of CFSE in PBS for 30 min at 37 °C and 5% CO_2_ and then washed twice in RPMI supplemented with 1% of FBS. Finally, we seeded 1 × 10^5^ cells/well in 6-well plates. At time 0 h, the just stained cells were analyzed in the flow cytometer (517 nm) alongside a set of unstained cells. Then, cells were exposed to 0.5, 1, or 2 µg/mL of fumagillin, or left untreated. The study was extended for 72 h, and daily samples were analyzed by flow cytometry. For that, cells were detached from each well using a cell scraper and resuspended in RPMI. The flow cytometer was set up to analyze 10,000 events from each sample, exciting the sample at 492 nm and measuring the fluorescence at 517 nm. In order to calculate the statistical differences between conditions, the average fluorescence of the cells without treatment was compared with the average fluorescence of the cells treated with the abovementioned fumagillin concentrations.

On the other hand, cell viability assays were carried out using Propidium Iodide Ready Flow™ Reagent (Invitrogen, Carlsbad, CA, USA) for 15 min following the manufacturer’s instructions. Cell density, detachment protocol, and the number of cells analyzed in each run were the same as in the proliferation assays. After cell staining, samples were excited at 535 nm, following the manufacturer’s instructions, and the emission was at 617 nm. Results were expressed as a percentage of cell viability.

### 2.12. Phagocytosis Assay and Fungal Growth

To analyze the interactions of the fungal strains with RAW 264.7 macrophages and BMMs, we seeded 2 × 10^5^ cells/mL in 500 µL of complete RPMI using 24-well plates (ThermoFisher) which contained 12 mm-diameter coverslips (ThermoFisher). After overnight incubation, immune cells were co-cultured with *A. fumigatus* conidia of each strain (Wt, ∆*fmaA*, ∆*fmaA*::*fmaA*) at a multiplicity of infection of 10 (ten conidia per immune cell). In parallel, we seeded the same number of each fungal strain conidia but in this case without immune cells to analyze fungal growth in the absence of murine cells. At each incubation time (2, 4, 6, and 8 h), we removed the coverslip to a new plate in order to calculate the percentage of phagocytosis, fungal germination, and hyphal branching. For that purpose, a minimum of 500 fungal cells was counted using a Nikon Eclipse TE2000-U inverted microscope.

Furthermore, we performed the same phagocytosis assay under two additional conditions. The first one, using RAW 264.7 macrophages pre-treated for 24 h with 1 µg/mL of fumagillin (before the co-incubation) to determine the effect of the toxin itself, and the second one using heat-inactivated conidia (121 °C for 30 min) of each *A. fumigatus* strain to ensure that the genetic manipulation of the strains did not produce structural changes of the conidia.

On the other hand, to study changes in the hyphal length of strains when growing alone or combined with either of the macrophage models, we performed CW staining on coverslips after 8 h of incubation. For that, samples were washed with PBS and stained with 100 µL of CW solution for 20 min in the dark and at room temperature. Finally, we washed the coverslips again with PBS, and they were mounted for fluorescence microscopy. Ten micrographs per coverslip were taken randomly using an Eclipse Ni microscope fluorescence microscope and a Nikon Ds-Fi2 camera (Nikon). Image analysis was carried out using ImageJ software.

### 2.13. Mouse Model of Pulmonary Aspergillosis

Mice used in this study were kept in the General Animal Unit Service of the University of the Basque Country (SGIker, UPV/EHU), with water and food ad libitum, handled in biological safety cabinets, and kept in sterilized cages with negative-pressure ventilation and filters.

SWISS female mice weighing 25–35 g were used in this study. All mice were immunosuppressed by the daily administration of 100 mg/kg cyclophosphamide (Sigma–Aldrich), starting four days before infection. Five animals per group were infected intranasally with 20 µL SS-T solution containing 1 × 10^7^ resting conidia of one *A. fumigatus* strain, either Wt, ∆*fmaA* or ∆*fmaA*::*fmaA*. Two independent experiments were performed.

Mice were monitored daily to analyze changes in weight and clinical scores due to the infection process. Clinical scoring was performed using a scale of 0–10, in which we evaluated different aspects of the disease (Table S1) following the recommendations of experts from our institution animal facility and the ethical committee members. Those mice that reached humane endpoints (10 points) were euthanized to minimize mice suffering, and their cause of death was considered as fungal infection.

To study the fungal burden in mice lungs, organs were extracted and were homogenized in 1 mL of SS-T by vortexing in a tube in the presence of a sterile glass stick. Finally, an aliquot of 0.1 mL was inoculated in potato dextrose agar plates supplemented with 10 μg/mL chloramphenicol and 25 μg/mL gentamicin (both from Sigma–Aldrich). Plates were incubated at 37 °C, and the number of Colony-Forming Units (CFUs) was determined after 3 days.

### 2.14. Statistics

All the assays were done at least by triplicate in three independent days. All statistical analyses of this study were performed using GraphPad Prism 7 software (GraphPad Software Inc., San Diego, CA, USA). At least three biological replicates were performed to measure each parameter in each experimental condition; any statistically significant differences were analyzed as required. All data present in this study followed a normal distribution. *t*-test or ANOVA was used to study differences between conditions depending on if we compared punctual data or multiple comparisons, respectively.

## 3. Results

### 3.1. Characterization of A. fumigatus Strains: Phenotype and Ability to Produce and Tolerate Fumagillin

To monitor any potential effect of loss of fumagillin synthesis in *A. fumigatus*, a phenotypic characterization following the spot dilution protocol was performed. Our results did not show any significant difference between the Wt, ∆*fmaA*, and ∆*fmaA*::*fmaA* strains under the different structural, osmotic, and oxidative stresses tested (Figure S1).

**Figure 1:**
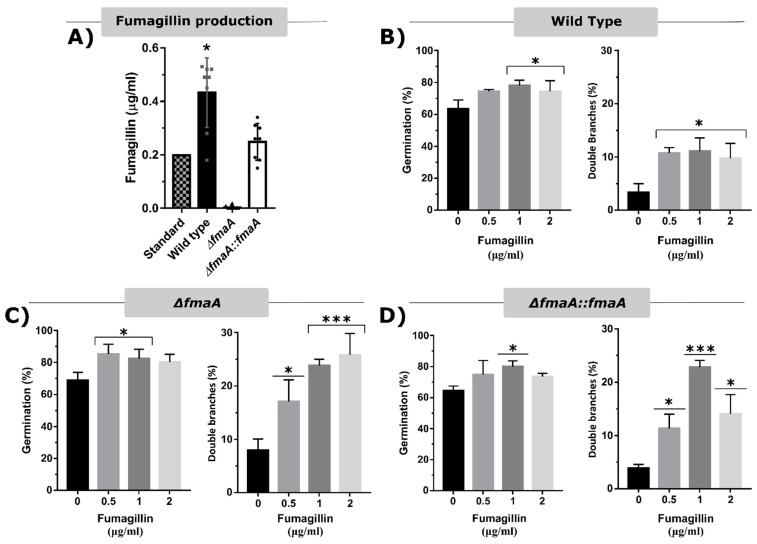
Fungal strains fumagillin production ability and self-resistance to the toxin. (**A**) Quantification of fumagillin secretion by the three *A. fumigatus* strains after 48 h of incubation in complete RPMI measured by UHPLC using commercial fumagillin (0.2 µg/mL) as an internal control. (* *p* < 0.05). (**B**–**D**) Germination and double branch assay of the Wt, Δ*fmaA*, and Δ*fmaA*::*fmaA*, respectively, after 8 h of exposition to 0.5, 1, and 2 µg/mL of fumagillin. All the experiments were done in triplicate in three independent assays. Significant differences respect to the control without the toxin are represented (* *p* < 0.05, *** *p* < 0.001).

The fungal strains’ ability to secrete fumagillin was also studied by UHPLC using a fixed concentration of toxin (0.2 µg/mL) as an internal control (Figure 1A). Consistent with previous results, the ∆*fmaA* strain was unable to produce fumagillin in vitro. In contrast, the Wt strain produced an average concentration of 0.43 µg/mL fumagillin (Figure 1B). The strain ∆*fmaA*::*fmaA* secreted an average concentration of 0.25 µg/mL fumagillin, although the concentration of fumagillin secreted was higher than that produced by the Wt in 25% of the assays. These results suggested great variability in fumagillin production by the WT and ∆*fmaA*::*fmaA* strains during growth.

In order to study any effect of exogenous fumagillin on *A. fumigatus*, a growth and germination assay of the Wt, ∆*fmaA*, and ∆*fmaA*::*fmaA* in the presence of 0.5, 1, and 2 µg/mL of fumagillin was performed. The results showed that fumagillin did not inhibit fungal growth (Figure S2) and that germination of the three strains was similar, being increased in the presence of the toxin (Figure 1B–D). Moreover, the addition of fumagillin was statistically correlated with the emergence of two germination tubes/conidium at 8 h of incubation. This effect was more pronounced on the fumagillin non-producer strain than on the Wt and ∆*fmaA*::*fmaA* strains (Figure 1B–D).

### 3.2. Different Fumagillin Uptake Ability in Cell Lines

**Figure 2:**
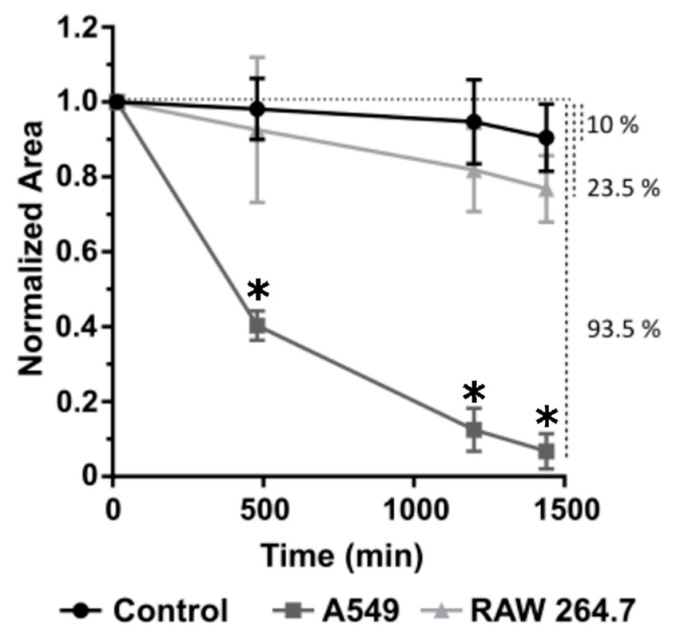
Quantification of the fumagillin absorption ability of each cell line. Fumagillin (1 µg/mL in complete RPMI) was added to empty wells (black line) or wells containing either A549 (dark grey) or RAW 264.7 cells (light grey), and its concentration was determined at 8, 20, and 22 h after inoculation by UHPLC. The total loss of fumagillin concentration after 24 h of exposition, compared to the initial concentration, is represented in percentage on the right axis of the graph (vertical dot lines). All the experiments were done in triplicate in three independent assays (* *p* < 0.05).

Since toxins need to be in contact with their target to fulfill their function, we measured fumagillin concentration dynamics by UHPLC in the culture medium when macrophages (RAW 264.7) or lung epithelial cells (A549) were exposed to the toxin for 24 h (Figure 2). Our data showed that about 10% of the fumagillin disappeared from the culture medium even in the absence of any of the cell lines, likely due to degradation processes. On the other hand, in the wells with macrophages, a higher progressive reduction in fumagillin concentration in the culture medium was detected, being 23.5% less after 24 h of exposition. More surprising were the results obtained in wells with epithelial cells, where a 93.5% of the initially added fumagillin was lost in the same period. Furthermore, we were able to recover fumagillin, 30 ± 14 µg/L and 21 ± 4 µg/L from the pellet of cells treated with RIPA buffer of A549 and RAW 264.7 culture, respectively, after exposure to 2 mg/L of fumagillin (Figure S3). No degradation forms of this molecule were detected either in the culture supernatant or in extractions of lysed cells.

### 3.3. Fumagillin Induces a Decrease in Cellular Activity and a Delay in Proliferation in Lung Epithelial Cells

**Figure 3:**
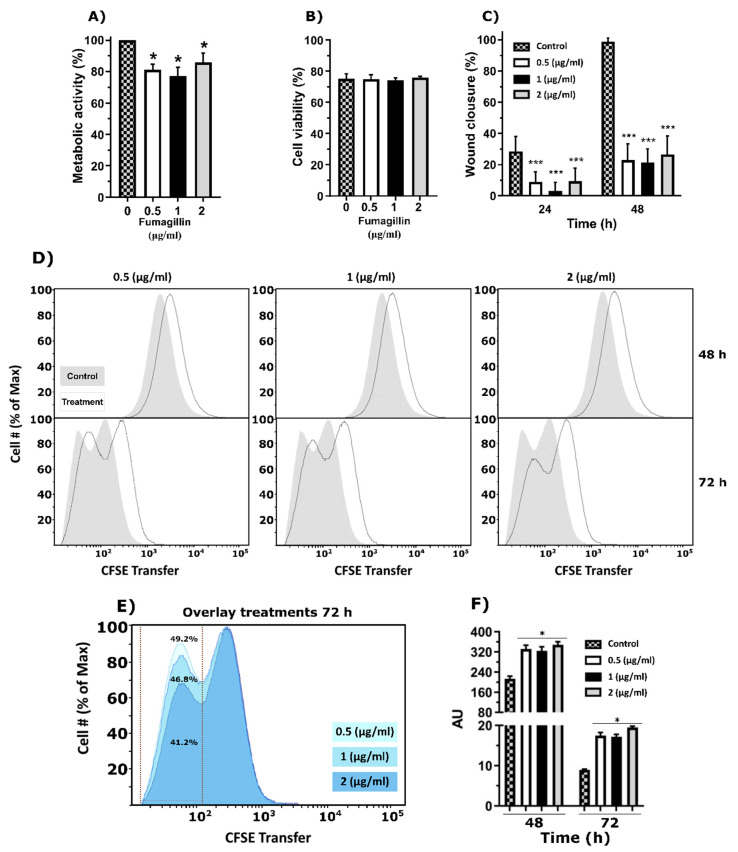
Fumagillin effect on A549 lung epithelial cells. (**A**) Electron transport chain activity of cell cultures growing in the presence of different fumagillin concentrations after 24 h of treatment. Results are expressed relative to cells growing without fumagillin. (**B**) Cell viability of propidium iodide-stained A549 cells measured by flow cytometry. (**C**) Wound closure of A549 cells when exposed to fumagillin for 24 and 48 h. (**D**) CFSE analysis of the A549 cell line in the presence of 0.5, 1, and 2 µg/mL of fumagillin after 48 and 72 h. Data are shown as the percentage of cells normalized versus the CFSE transferred. The grey areas represent the control cells without fumagillin, while the black line represents the cells after exposition to the mycotoxin. (**E**) Overlay results obtained at 72 h with the three concentrations of fumagillin tested. (**F**) Fluorescence intensity of the CFSE results after 48 and 72 h of treatment with fumagillin. In CFSE terms, more fluorescence means less cell proliferation. All the experiments were done by triplicate in three independent assays. (**A**–**C**,**F**) Data were analyzed by one-way ANOVA with respect to controls without fumagillin. (* *p* < 0.05; *** *p* < 0.001).

To evaluate the effect of fumagillin on the electron transport chain (ETC) activity of A549 epithelial cells, MTT reduction assays were performed (Figure 3A). ETC activity was lower in cells growing for 24 h in the presence of fumagillin at any of the concentrations tested in comparison with those growing without fumagillin. In contrast, the staining with propidium iodide showed no changes in cell viability, with about 75% of cells being viable after 24 h of incubation in all conditions tested (Figure 3B).

The proliferation and migration abilities of A549 cells in the presence of the toxin were also studied using the wound healing assay. Notably, fumagillin inhibited the ability of epithelial cells to close the gap caused in the monolayer by a pipette tip after 24 h of incubation with all the fumagillin concentrations tested. The results were even more pronounced after 48 h of incubation (Figure 3C), where only a wound closure of around 20% was measured in the cultures with the mycotoxin, while in control cells, it was completely closed.

Finally, cell proliferation was studied by flow cytometry in cells exposed to fumagillin, showing a similar delay after 48 h of exposition to all the fumagillin concentrations tested (Figure 3D). In contrast, after 72 h, a marked fumagillin concentration-dependent effect was detected. At this time, in both control and treated cells, two peaks of fluorescence were observed, corresponding to two different cell generations. The overlay graph with the different concentrations of fumagillin (Figure 3E) demonstrates that exposure to the toxin was critical for the development of the second generation of cells. In fact, the number of cells detected in this phase when they were incubated with 2 µg/mL of fumagillin was 8% less than with 0.5 µg/mL. Finally, it is important to highlight that the differences shown in the cell proliferation panel (both after 48 and 72 h of treatment) (Figure 3D) were statistically significant when the average fluorescence of each condition was compared (Figure 3F).

### 3.4. Fumagillin Reduces Cellular Activity and Viability of Macrophages

**Figure 4:**
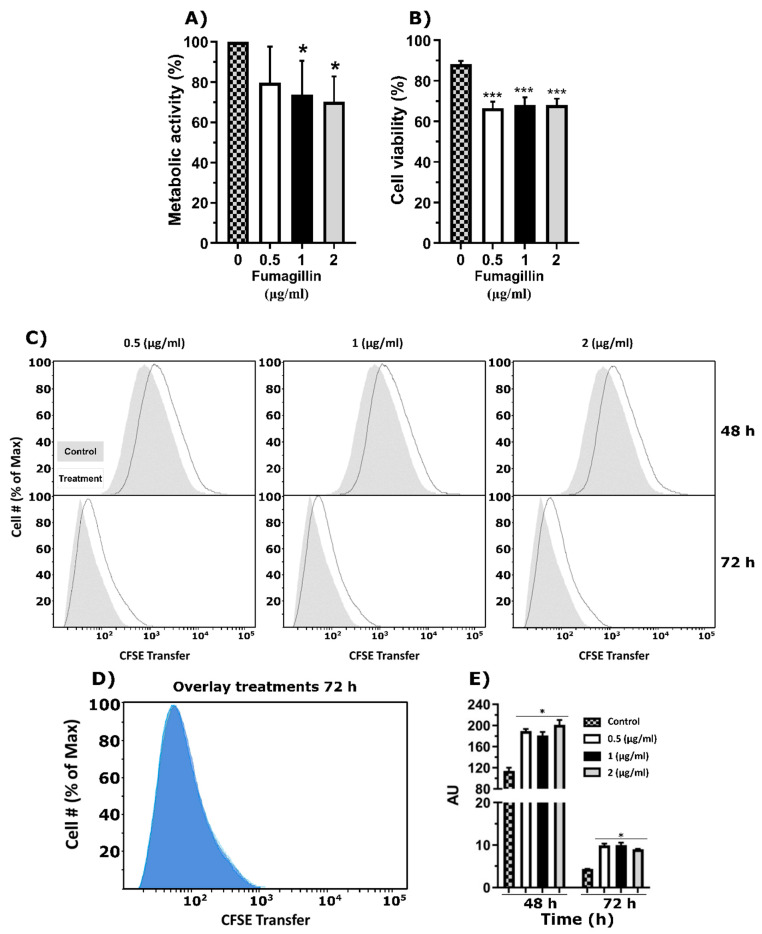
Fumagillin effect on RAW 264.7 macrophages. (**A**) Cell activity of the cell cultures growing in the presence of different fumagillin concentrations for 24 h. Results are expressed relative to the cells growing without fumagillin. (**B**) Cell viability of propidium iodide-stained RAW 264.7 cells measured by flow cytometry. (**C**) CFSE analysis of RAW 264.7 cells exposed to 0.5, 1, and 2 µg/mL of fumagillin for 72 h. The percentage of cells normalized versus the CFSE transferred at 48 h and 72 h is plotted. The grey areas represent the control cells without fumagillin, while the black lines represent the results of the cells after exposition to the mycotoxin. (**D**) The last panel shows the overlay results obtained at 72 h with the three concentrations of fumagillin tested. (**E**) Fluorescence intensity of the CFSE results after 48 and 72 h of treatment with fumagillin. In CFSE terms, more fluorescence means less cell proliferation. All the experiments were done in triplicate in three independent assays. (**A**,**B**,**E**) Data were analyzed by one-way ANOVA respect to controls without fumagillin. (* *p* < 0.05; *** *p* < 0.001).

Similar to what we observed in epithelial cells, the ETC activity of RAW 264.7 macrophages were significantly lower after 24 h of incubation with 1 and 2 µg/mL of fumagillin in comparison with those without mycotoxin and even with 0.5 µg/mL (Figure 4A). Moreover, the propidium iodide assay showed a significant reduction in cell viability in the presence of all the fumagillin concentrations used (Figure 4B).

The proliferation analysis also showed a slight delay in cell proliferation after 48 and 72 h of toxin exposition in comparison with the control cells without fumagillin (Figure 4C), but this effect was not concentration-dependent (Figure 4D). As happened with A549 cells, the differences shown in the cell proliferation panel (Figure 4C)) were statistically significant when the average fluorescence of each condition was compared (Figure 4E).

### 3.5. The Loss of the Ability to Produce Fumagillin Is Associated with a Transient Higher Rate of Phagocytosis of A. fumigatus Conidia

**Figure 5:**
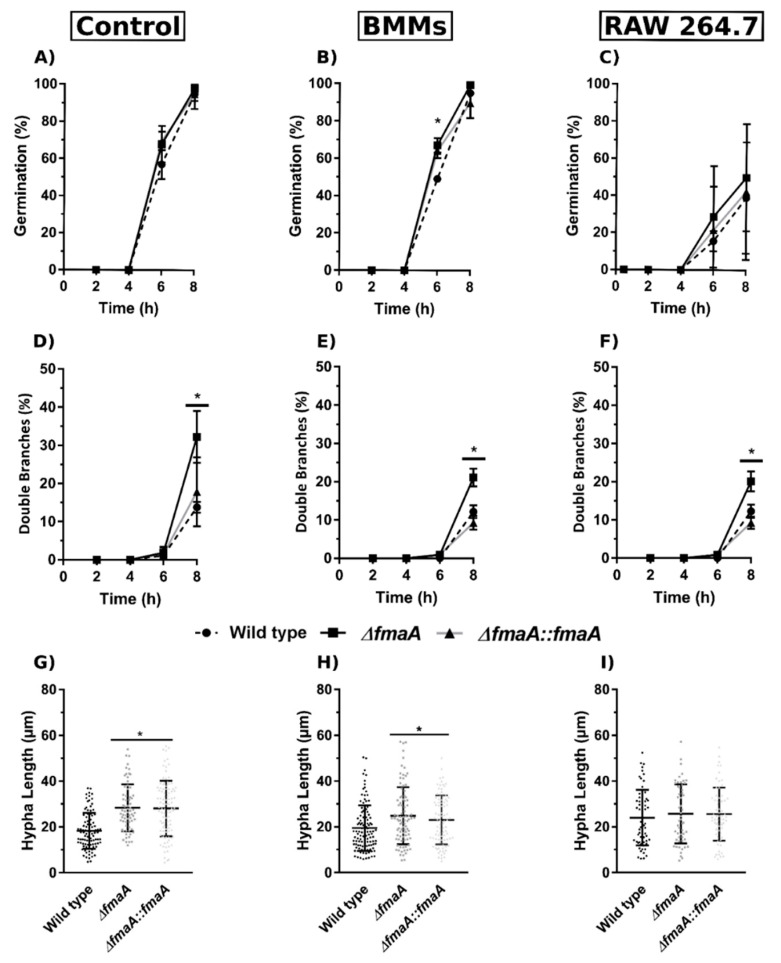
Growth dynamics of fungal strains in co-incubation with macrophages. Data of fungal germination (%), double branch germination (%), and hyphal length after 8 h of incubation of the three fungal strains growing either alone (**A**,**D**,**G**) (Control), in co-culture with BMMs (**B**,**E**,**H**), or in co-culture with RAW 264.7 macrophages (**C**,**F**,**I**). * *p* < 0.05.

In order to understand whether fumagillin secretion can alter the interaction between the fungus and macrophages, we performed co-cultures using the three fungal strains and either BMMs or RAW 264.7 cells and we analyzed the fungal germination and branching rates, as well as the phagocytosis index. Concerning germination, no differences between strains were found neither in control nor when the fungus was in contact with RAW 264.7 cells. However, in the presence of BMMs, the germination rates of ∆*fmaA* and ∆*fmaA*::*fmaA* strains were significantly higher than those of Wt at 6 h, reaching the same value at 8 h in all the strains (Figure 5A–C). Moreover, the development of the second branch of the hyphae of the fumagillin non-producer strain was significantly higher than those observed for Wt and ∆*fmaA*::*fmaA* when exposed to macrophages (Figure 5D–F) as observed previously in fumagillin contact studies.

Regarding hyphal length, the ∆*fmaA* and ∆*fmaA*::*fmaA* strains grew significantly longer hyphae than the Wt when the fungus grew alone or in co-culture with BMMs (Figure 5G,H). On the other hand, no differences in hyphal length were found between strains when they grew in the presence of RAW 264.7 macrophages (Figure 5I).

**Figure 6:**
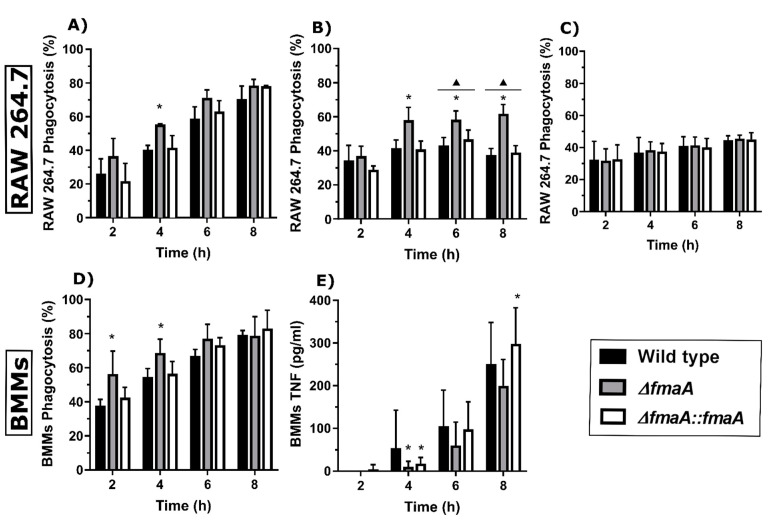
Phagocytosis dynamics in fungus–macrophage co-cultures. (**A**) Phagocytosis results of co-incubation of each fungal strain and the macrophage cell line RAW 264.7. (**B**) Phagocytosis results of macrophages RAW 264.7 pre-treated with 1µg/mL of fumagillin for 24 h and then co-incubated with each fungal strain. After the treatment time, the RPMI medium with fumagillin was replaced with fresh complete RPMI. (**C**) Phagocytosis results of co-incubation between heat-inactivated conidia of the three fungal strains and RAW 264.7 cells. (**D**) Phagocytosis results of co-incubation of the three fungal strains and BMMs. (**E**) TNF production by BMMs during co-incubation with each fungal strain. All the experiments were done in triplicate in three independent assays. In each case and time point, co-cultures of Wt strain were used as control (* *p* < 0.05). In the case of panel (**B**), a solid triangle means *p* < 0.005 in comparison with the same time point in panel (**A**). Comparisons between different time points were not plotted.

Finally, we analyzed the macrophage phagocytosis of fungal cells for 8 h and observed that the ∆*fmaA* strain was phagocytosed at a significantly higher rate during the first 4 h of contact with both RAW 264.7 and BMMs cells (Figure 6A,D, respectively). However, there were no significant differences in the phagocytosis at 6 and 8 h. The pre-treatment of RAW 264.7 cells with fumagillin before the co-incubation with the fungal strains only showed a significant increase in phagocytosis of the non-fumagillin producer strain (Figure 6B). Comparing the untreated assay (Figure 6A) with the pre-treated assay (Figure 6B), we found that the pre-treated cells showed significantly lower phagocytosis rates after 6 and 8 h of co-incubation (solid triangles). This may imply that macrophages were affected by the pre-treatment, showing lower phagocytic ability. The phagocytosis study performed using heat-inactivated conidia of the three strains did not show differences between these fungal strains (Figure 6C). As the phagocytosis rate of the ∆*fmaA* strain by BMMs was the most efficient during the first 6 h (Figure 6D), we studied TNF production in these co-cultures. Overall, there was a trend showing that the ∆*fmaA* strain stimulated a lower TNF release by BMMs in all the time points analyzed, although a great biological variability between replicates was observed. Specifically, at 4 h, ∆*fmaA* and ∆*fmaA*::*fmaA* showed a significantly lower capacity to induce TNF production by BMMs than the Wt. The significantly higher production of TNF by the BMMs in contact with ∆*fmaA*::*fmaA* compared to the Wt and ∆*fmaA* strain after 8 h of co-incubation (Figure 6E) was surprising.

### 3.6. Fumagillin Synthesis Increases Fungal Load during Infection

**Figure 7:**
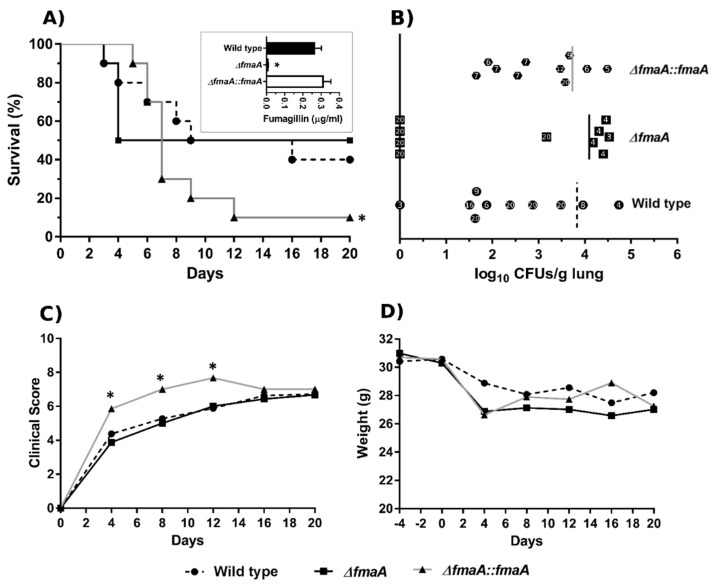
Intranasal infection of a leukopenic mouse model with *A. fumigatus*. (**A**) Kaplan–Meier analysis of infected mice during 20 days post intranasal exposition to Wt, ∆*fmaA*, and ∆*fmaA*::*fmaA*. The panel also shows the fumagillin production ability of the conidia used to inoculate the mice. (**B**) Fungal burden analysis of mouse lungs. The numbers inside the symbols represent the death day of each mouse; day 20 indicates survival at the end of the study period. Vertical lines show 50% mortality in each case. (**C**) Values of the clinical score throughout the experiment. (**D**) Evolution of the mice weight during the experiment. All the experiments were done in triplicate in three independent assays. Data of Wt strains were used as control (* *p* < 0.05).

To evaluate the importance of fumagillin production for *A. fumigatus* virulence in vivo, we used a mouse model of pulmonary aspergillosis already established in our lab [23]. Figure 7A shows that the ∆*fmaA* strain induced a 50% mortality in the first 4 days post-infection, but mice that survived after this day remained alive throughout the experiment. In contrast, the mice infected with the Wt showed a more progressive increase in mortality rate reaching 60% at the end of the experiment. Finally, it is important to highlight that the ∆*fmaA*::*fmaA* strain was significantly more lethal than the Wt and ∆*fmaA* strain. Considering the biological variability observed in the concentration of fumagillin secreted by the strains, the conidia suspensions used for the infections were for their specific ability to produce fumagillin by UHPLC at the same time. This revealed that the ∆*fmaA*::*fmaA* cells used in these in vivo experiments were able to produce more fumagillin than the Wt strain, but the differences were not statistically significant. The ∆*fmaA* strain used did not produce fumagillin, as expected.

The fungal burden was slightly higher in mice infected with the ∆*fmaA* than in the others (Figure 7B). However, the average fungal burden of the mice infected with ∆*fmaA* was measured from the 50% of mice died during the first four days, which presented high CFUs values because, surprisingly, in the lungs of almost all the mice (4 out of 5) that survived the experiment, CFUs were not found. In contrast, mice infected with the Wt and the ∆*fmaA*::*fmaA* strains presented variable CFUs counts without a clear relationship between the day of the death and the fungal burden, and even survivor mice presented high CFUs values, except for one.

Finally, the symptoms of the infected mice were monitored using a scale of 0 to 10, showing that mice infected with the ∆*fmaA*::*fmaA* strain presented significantly more severe symptomatology than those infected with the Wt and the ∆*fmaA* strain (Figure 7C). From day 16 to the end of the experiment, the clinical score values were similar between all the groups. In contrast to the clinical score, mice weight (Figure 7D) was the same regardless of the fungal strain used to infect the mice.

## 4. Discussion

The importance of gliotoxin for *A. fumigatus* virulence has been widely described [12,13,14,15,30,31,32,33]. However, the relevance of other toxins produced by *A. fumigatus*, such as fumagillin, has not been studied in depth.

Fumagillin is a macrolide that targets methionine aminopeptidase 2 (MetAP2) by covalent binding to the His231, inhibiting the proper functioning of the protein. Recent publications have pointed out that fumagillin could induce lung epithelial cell damage, both acting alone [23] and in synergy with gliotoxin [34]. Moreover, this mycotoxin is able to inhibit cell proliferation and angiogenesis in endothelial cells [20]. However, little is known about its effect on the fungus or other cell types or its role in fungal virulence in vivo during the development of pulmonary aspergillosis.

To study this, we used a deletion mutant strain of the *fmaA* gene and its complement strain where *fmaA* encodes a terpene cyclase required for fumagillin synthesis [22]. First, an UHPLC method for the detection of fumagillin in samples of RPMI obtained after in vitro assays was standardized. Using this method, we were able to detect as low as 0.1 µg/mL of fumagillin. Furthermore, we confirmed that the ∆*fmaA* strain was not able to secrete fumagillin, corroborating the previously published data [22].

Then, we performed a macroscopic phenotypical and growth study that showed that the mutant strain ∆*fmaA* did not present any difference compared to the Wt strain when they grew in the presence of structural, osmotic, and oxidative stresses (Figures S1 and S3). We also observed that *A. fumigatus* was not susceptible to exogenous fumagillin (Figure S2), but that fumagillin may be a germination-stimulating factor (Figure 1). These results could support our hypothesis that the gene Afu8g00410, encoding a methionine aminopeptidase located inside the fumagillin cluster, confers resistance to the fungus. This gene would be co-expressed with the rest of the cluster, providing a resistant MetAP2 to the fungus and may explain the results obtained in Figure 1. Continuing with fungal strains’ germination ability, it is notable that the ∆*fmaA* strain reached higher germination and branching rates than the other strains growing without cells and in the presence of fumagillin. The increased branching rates observed in the ∆*fmaA* strain may indicate that fumagillin could be involved in a polarization process, or in contrast, the increased branching observed could be a consequence of the adaptive process to the fumagillin presence. In any case, both hypotheses should be studied in depth.

Analysis of the effect of synthetic fumagillin on both A549 pneumocytes and RAW 264.7 macrophages, which are common cell lines to perform toxin/molecule/drug response analysis [23,34,35], demonstrated that each cell type uptakes the mycotoxin in different ways (Figure 2). Fumagillin was clearly depleted from the supernatant (93.5%) of pneumocytes but to a much lesser degree (23.5%) macrophages culture. Furthermore, the lysis of both cell types using RIPA buffer and subsequent detection of the toxin by UHPLC suggested that the reduction in fumagillin from the media was due to its uptake by the cell. One explanation could be attributed to the different expression levels of the MetAP2 enzyme between both cell types (https://www.proteinatlas.org; accessed 15 August 2021). Moreover, we cannot forget that macrophages can express different efflux systems that could expel the toxin from the cytosol. Finally, we cannot totally discard that cells could also degrade fumagillin, but no peaks similar to the ones observed after the fumagillin degradation assays (light, temperature, and acid environment) were detected either in the supernatants or the pellets (Figure S2). Therefore, it seems that fumagillin could passively cross the cell membrane reaching the cell cytosol [36] and bind to MetAP2, thus leading to loss of the toxin in the supernatant.

The MTT results shown in this study are in accordance with the ones previously published [34,37,38], but our interpretation of the MTT results varies with the other studies. In this study, we used the MTT assay as a technique suitable to measure the ETC [39], while other authors used it to measure cell viability. On the other hand, it is important to note that our highest fumagillin concentration used (2 µg/mL) was the lowest of all these above-cited studies. In contrast, it is important to note that during the infection process in vivo or during the contact between the fungus and host cells, the toxin concentration reached could be higher. For instance, the production of candidalys in by *Candida albicans* in the invasion pocket produced an extremely high concentration of the toxin at the contact site between the fungus and the cell [40]. This hypothesis could explain why a previous study performed with this same ∆*fmaA* strain demonstrated that it was less toxic than the Wt to the A549 cell line as assessed by ^51^Cr release assays [23].

Our results support the affectation of the ETC activity, as both the wound healing assay and the CFSE analysis performed by flow cytometry demonstrated that the proliferation of epithelial cells was affected after fumagillin exposure but not its cellular viability. Specifically, the covalent binding of fumagillin to its molecular target, MetAP2, results in an inhibition of all functions that MetAP2 orchestrates inside the cell [21]. They include the membrane signaling pathway involving proteins, such as Gi/o, Giβγ, PI3K, PLC, DAG, or IP3 that govern cell migration and proliferation [41,42,43].

The results obtained with the RAW 264.7 cells were slightly different from those obtained from the pneumocytes since although they suffered a drop in the ETC activity and a delay in the cell proliferation after exposure to fumagillin, the effect was less marked than observed in pneumocytes and was not dependent on concentration. These results are apparently in contradiction with previous studies of other authors on rat alveolar macrophages using trypan blue to measure cell viability, which is a less sensitive method [44]. Currently, it is unknown why macrophages, but not epithelial cells die at such a high rate; the different effects of the fumagillin depending on the cell type might be explained because the MetAP2 concentration could vary among tissues and cell types (https://www.proteinatlas.org; accessed 15 August 2021).

In addition, fumagillin seems to protect, at least in early hours, the fungus from phagocytosis as assessed from increased phagocytosis of ∆*fmaA* conidia. In fact, when RAW 264.7 phagocytic cells were pre-treated with fumagillin, the phagocytic ability of these cells was significantly decreased after 6 and 8 h of co-incubation in comparison with the phagocytosis rates at this same time points of the cells untreated. Furthermore, the phagocytosis of the cells pre-treated was still significantly higher in contact with ∆*fmaA* strain at all times. These results, alongside the evidence that phagocytosis of the heat-inactivated conidia is similar in mutants and Wt strains, points out that the inhibition of the phagocytosis observed is because of the toxin effect over the macrophages and not because of an external structure defect of the conidia cell wall produced as a consequence of the genetic manipulation of the fungal strains. Moreover, it is important to note that the fumagillin produced by the Wt and complemented strains could be modulating the macrophages’ ETC and viability influencing the cell response, from their phagocytosis ability to TNF production. Indeed, TNF induction by BMMs was also overall lower in response to the ∆*fmaA* strain than to the Wt and complemented strains, although it was not statistically significant except at 4 h.

Regarding in vivo assays, Liu and coworkers evaluated the virulence ability of the mutant *A. fumigatus* non-fumagillin producer strain Δ*fumR* [24]. For that, they used a corticosteroid immunosuppressed mice model, and they did not describe any virulence difference between the mutant and the Wt strain. Therefore, and due to the differences in the phagocytosis assays observed (Figure 6), we wanted to assess a neutropenic murine aspergillosis model to look at the role of the *fmaA* gene in virulence. Unexpectedly, the ∆*fmaA*::*fmaA* strain was significantly more lethal than the Wt and ∆*fmaA* strains. In contrast, the lowest mortality rate (although not significantly different to the Wt) was observed in those mice infected with the ∆*fmaA* strain.

On the other hand, the CFUs analysis showed that most of the surviving mice infected with ∆*fmaA* strain did not contain viable fungus in their lungs, whereas a high fungal burden was detected in mice infected with the other strains at the end of the experiment. These results may indicate that the mortality caused by ∆*fmaA*, only observed during the first four days of infection, could be the result of the ability of this mutant to germinate faster than the others. Furthermore, the possible production of other virulence factors, such as proteases or other toxins, could be compensating for the lack of fumagillin, but this hypothesis has not been analyzed in this study.

To our knowledge, this is not the first fumagillin research, but it is the first in-depth study of the role of fumagillin on *A. fumigatus* virulence, showing that this molecule can affect host cell homeostasis and that *A. fumigatus* shows an intrinsic resistance to its own toxin. Finally, in our murine model, whereas we found that mice infected with ∆*fmaA* survived with lower fungal burden than those infected with the wild type or the complemented strain, there was little overall difference in virulence dependent on this toxin.

## Supplementary Materials

The following are available online at https://www.mdpi.com/article/10.3390/jof7110936/s1, Figure S1: Phenotypic characterization of fumagillin mutant strains; Figure S2: Growth curves of the fungal strains after exposition to 0, 0.5, 1, and 2 µg/mL of fumagillin for 70 h; Figure S3: Fumagillin detection and its degradative products by UHPLC; Figure S4: Inhibition assay of the fungal strains exposed to 30%, 15%, 7.5%, and 3.75% of H_2_O_2_ for 48, 75, and 96 h; Table S1: Clinical scoring table.
