# Supplementary material for: Aspergillus fumigatus Fumagillin Contributes to Host Cell Damage
Source: J Fungi (Basel). 2021 Nov 3;7(11):936. doi: 10.3390/jof7110936 (PMC8619997; doi:10.3390/jof7110936)
Supplement: Supplementary file 1 [file jof-07-00936-s001.zip › jof-1392486-supplementary/Supplemetary Figures.pdf]

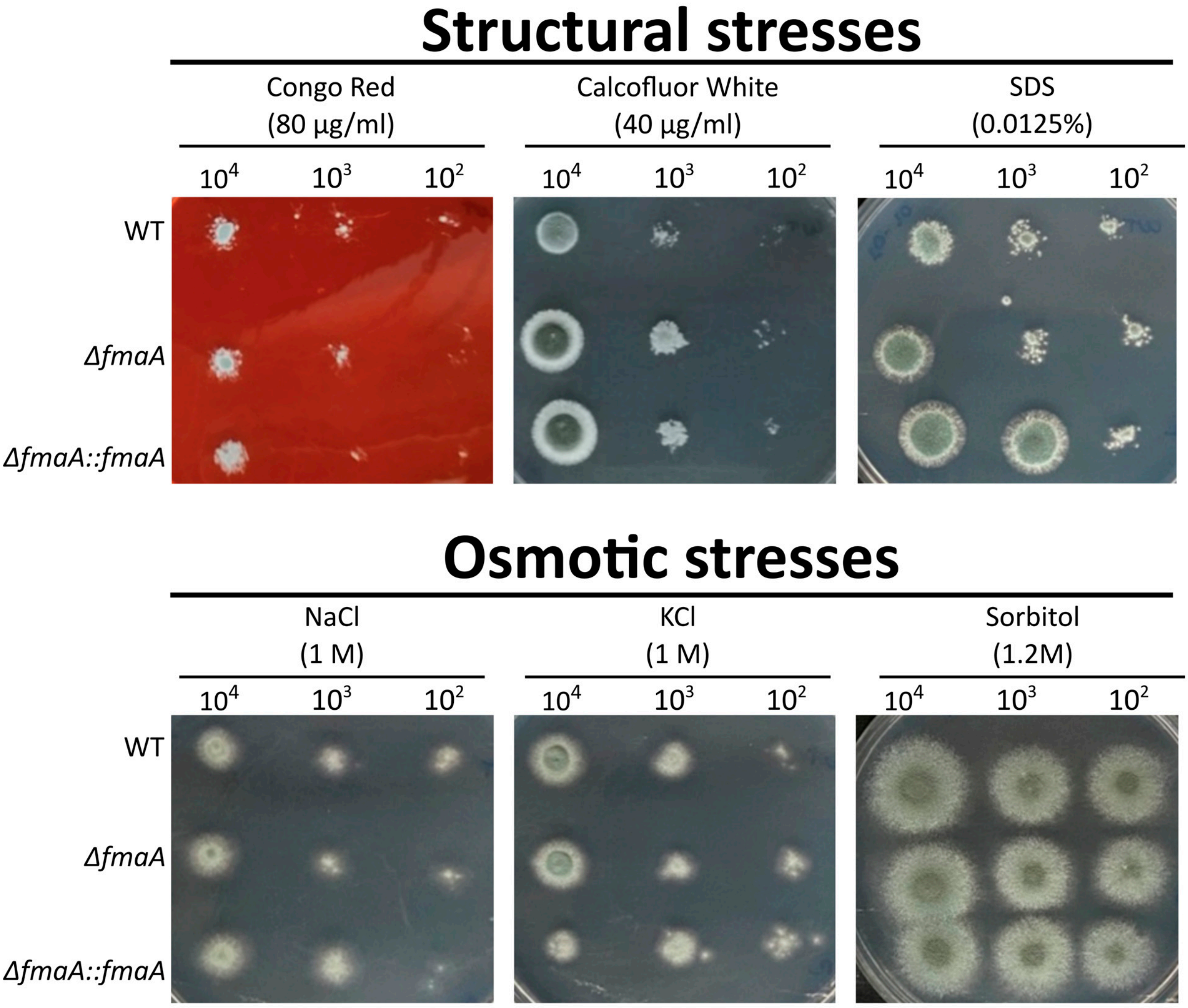

Figure S1. Phenotypic characterization of fumagillin mutant strains. Spot dilution assay of the three fungal strains (Wt, *ΔfmaA* and *ΔfmaA::fmaA)* growing in the presence of 80 µg/ml Congo Red, 40 µg/ml Calcofluor White, 0.0125% SDS, 1 M NaCl, 1 M KCl and 1.2 M Sorbitol. No growth differences between the strains were detected after phenotypic assays.

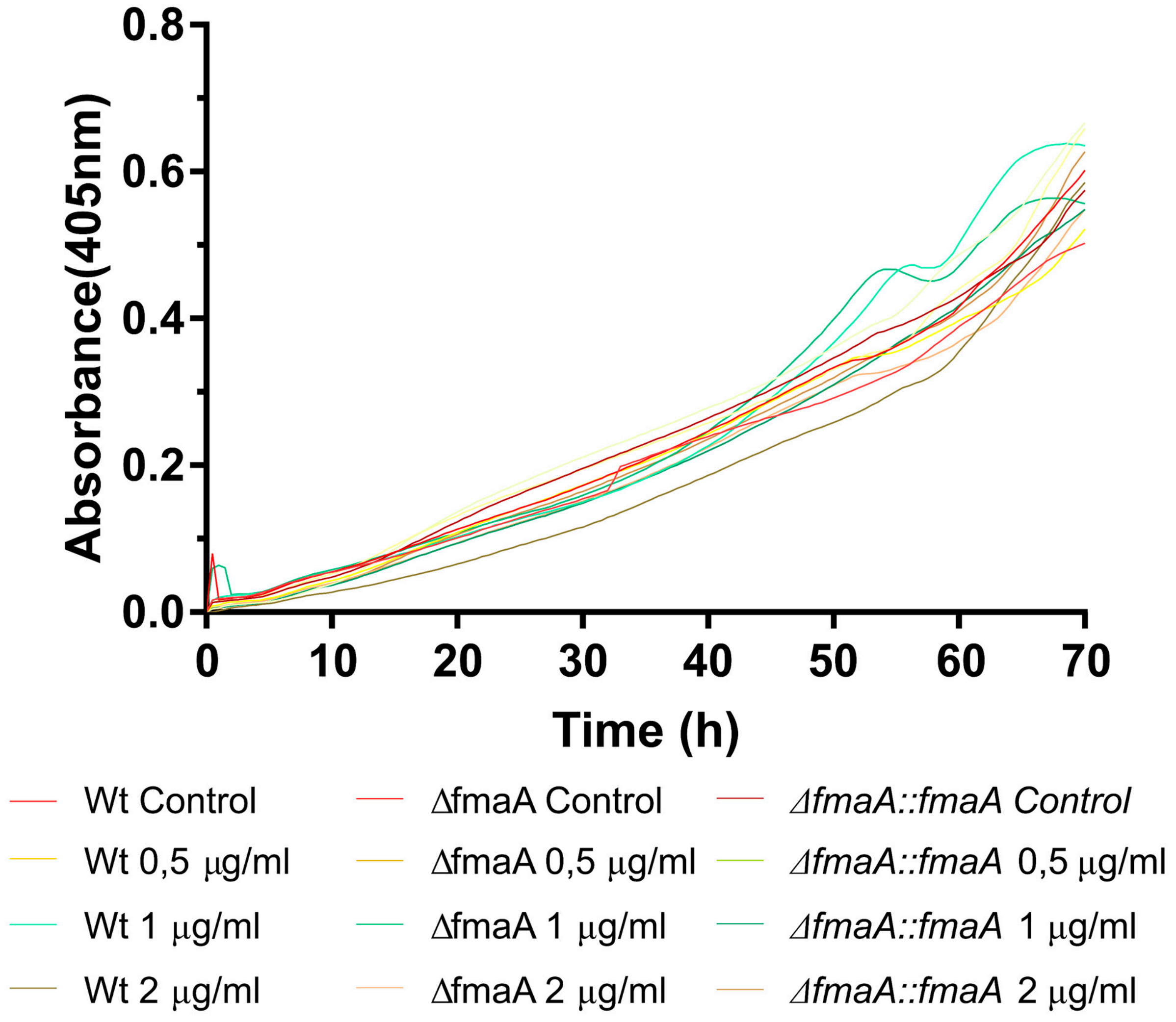

Figure S2. Growth curves of the fungal strains after exposition to 0, 0.5, 1 and 2 μg/ml of fumagillin for 70 hours. All the experiments were done by triplicate and the absorbance was measured at 405 nm. Due to the great amount of conditions/strains used, only the mean values (without standard errors) are plotted.

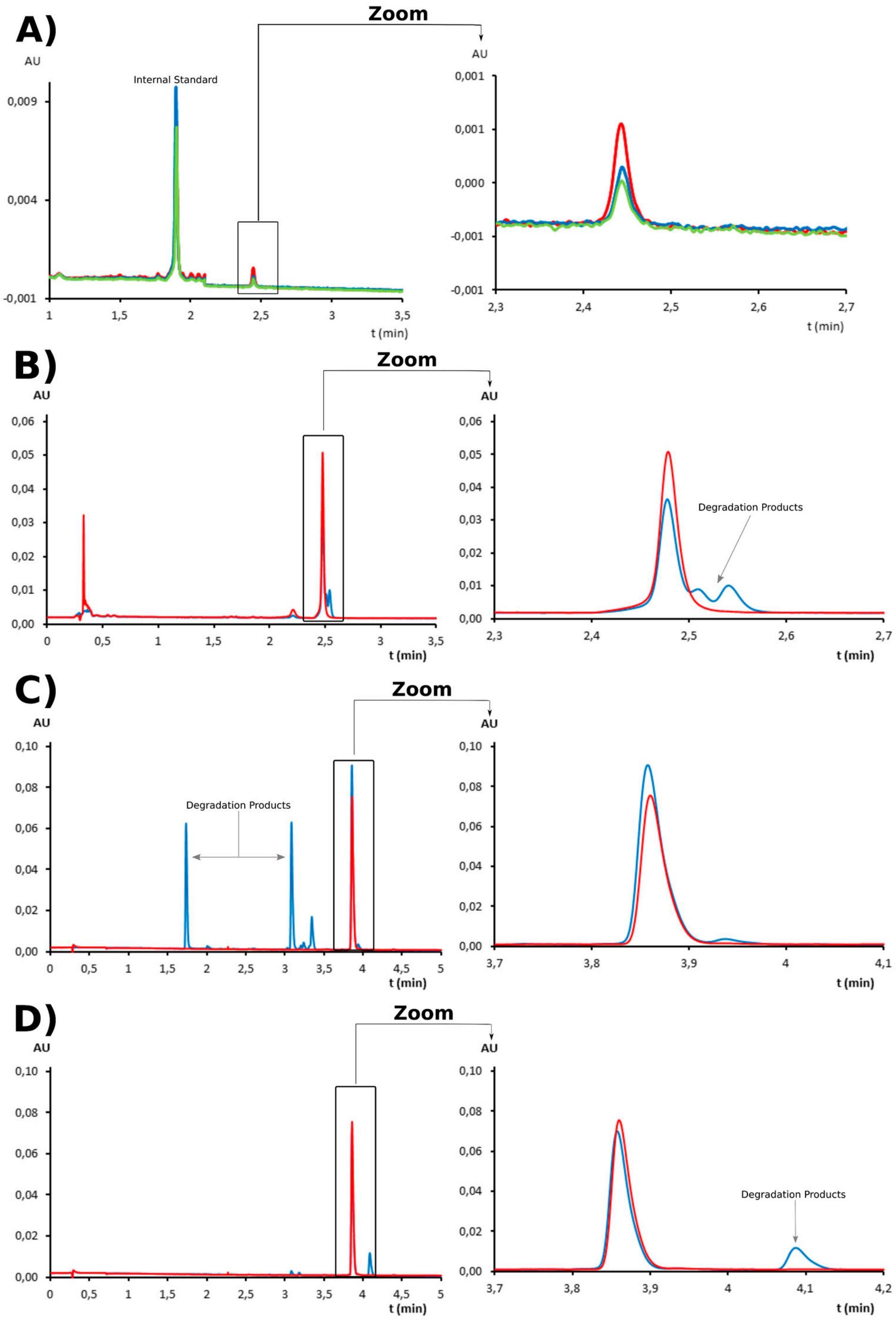

Figure S3. Fumagillin detection and its degradative products by UHPLC. (**A**) Chromatograms after treatment of A549 and RAW 264.7 cell cultures with RIPA buffer. Cell cultures grew for 24 hours in complete medium supplemented with 2 μg/ml of fumagillin before RIPA treatment. Red: Standard; Blue: A549; Green: RAW 264.7. Diclofenac was used as an internal standard. (**B**) Results obtained after fumagillin light exposure for two hours. Red: Fumagillin without light treatment; Blue: Fumagillin treated with light. (**C**) Results obtained after fumagillin incubation at 80°C for 22 hours. Red: Fumagillin without treatment; Blue: Fumagillin treated. (**D**) Results obtained after fumagillin exposition to pH 1 for 22 hours. Red: Fumagillin without treatment; Blue: Fumagillin treated.

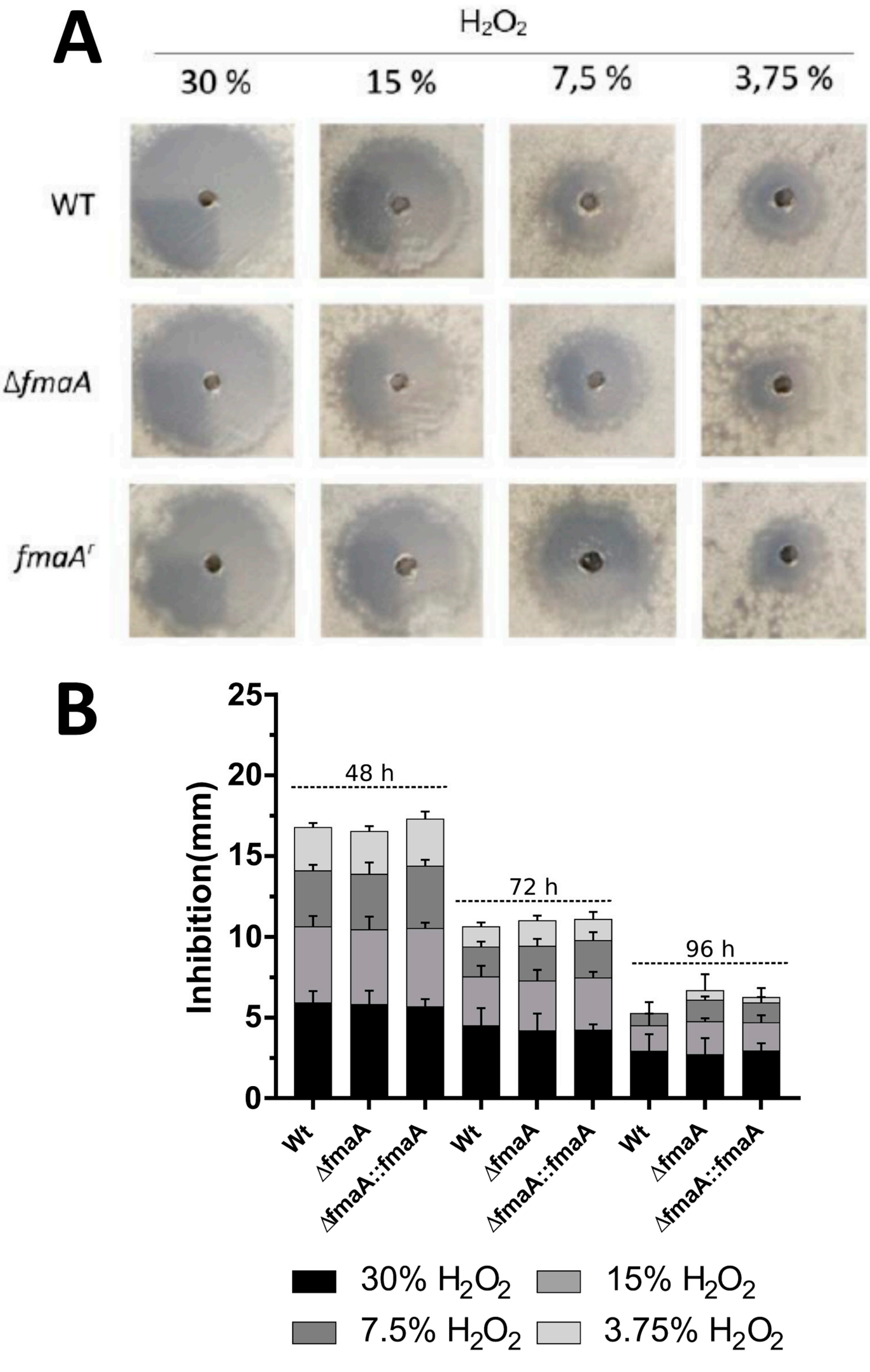

Figure S4. Inhibition assay of the fungal strains exposed to 30%, 15%, 7.5% and 3.75% of H_2O_2 for 48, 75 and 96 hours. (**A**) Inhibition halos of the different strains in cultures; (**B**) Culture inhibition measurements (mm).
